# Efficiency assessment of intelligent patient-specific instrumentation in total knee arthroplasty: a prospective randomized controlled trial

**DOI:** 10.1186/s13018-024-05010-5

**Published:** 2024-09-28

**Authors:** Guoqing Liao, Jinmei Duoji, Lishuai Mu, Yiling Zhang, Xingyu Liu, Daozhang Cai, Chang Zhao

**Affiliations:** 1 Department of Joint Surgery, Center for Orthopedic Surgery, The Third Affiliated Hospital of Southern Medical University, Guangzhou, Guangdong China; 2 Department of Orthopedics, Nyingchi People’s Hospital, Nyingchi, Xizang China; 3 School of Biomedical Engineering, Tsinghua University, Beijing, China; 4 Longwood Valley Medical Technology Co. Ltd, Beijing, China; 5 School of Life Sciences, Tsinghua University, Beijing, 100084 China

**Keywords:** TKA, Patient-specific instrumentation, Artificial intelligent, Resection accuracy, Alignment

## Abstract

**Background:**

In total knee arthroplasty (TKA), the practical use of patient-specific instrumentation (PSI) has been reported previously with both advantage and disadvantage. The application of artificial intelligent (AI) forces overwhelmingly development of medical industries, while the impact of AI on PSI efficiency remains unknown. Thus, this study aimed to assess the efficiency of Intelligent-PSI (i-PSI) in TKA, compared with the conventional instrumentation-TKA (CI).

**Methods:**

102 late-stage OA patients who met inclusive criteria were recruited in this prospective randomized controlled trial and separated into two groups (i-PSI vs. CI). In both groups, an AI preoperative planning engine was applied for surgery decision making. In CI group, conventional instrumentation was applied for bony resection, while resection of i-PSI group was completed with i-PSI. A convolutional neural network was applied to automatically process computer tomography images and thus produced i-PSI. With the help of three-dimension printing, the workflow of production was largely simplified. AI-driven preoperative planning guided resection and alignment decisions. Resection measurement, perioperative radiography and perioperative clinical outcomes were analyzed to verify efficiency of i-PSI.

**Results:**

In resection outcomes, smaller deviation of lateral and medial distal femoral resection were found in i-PSI group than CI group (*P* = 0.032 and 0.035), while no difference was found in other resection planes. In radiography outcomes, postoperative coronal alignments of i-PSI group, including postoperative Hip–knee–ankle axis (HKA) (*P* = 0.025), postoperative HKA outliners (*P* = 0.042), Femoral coronal alignment (FCA) (*P* = 0.019) and Joint line convergence angle (JLCA) (*P* = 0.043) showed closer to neutral position than CI group. Moreover, Femoral sagittal alignment (FSA) of i-PSI group showed closer to neutral position than CI group(*P* = 0.005). No difference was found in other alignments. In clinical outcomes, i-PSI group seemed to cost more surgical time than CI group (*P* = 0.027), while others showed no differences between the two groups.

**Conclusion:**

Intelligent Patient-specific Instrumentation in TKA achieved simplified production flow than conventional PSI, while also showed more accurate resection, improved synthesis position and limb alignment than conventional instrumentation. Above all, this study proved that i-PSI being an applicable and promising tool in TKA.

## Introduction

Knee osteoarthritis (KOA) is a degenerative whole joint disease involving all knee tissues, which causes burdensome on the health and economies, affecting millions of people. Total knee arthroplasty (TKA), one of the most clinically successful medical procedures [1, 2], has been deemed as an optimal treatment to end-stage KOA. As the operation develops, the quest of minimizing damages and optimizing outcomes accelerates new techniques to emerge and emphasizes the combination of medicine and engineering [3].

In TKA, conventional instrumentation (CI), which stands for supporting tools supplied by prosthesis kits, confronts the threat of robotic technology. Robotic TKA not only improve the accuracy of implant positions and functional outcomes but also reduce the damages of periarticular soft tissue [4, 5]. But the drawbacks, including costs, learning curve and complications, limit the practicability of Robotic TKA especially in low volume surgical centers [6–9].

Nevertheless, Patient-specific instrumentation (PSI), a resection guide designed basing on preoperative CT or MR images and aimed to set personal resection plan for diverse people, shows better practicability and no limitations of costs or learning curve even in low volume surgical centers [10]. Supporters have proven that PSI has advantages of improved alignments, reduced surgical time and better clinical outcomes, compared to the conventional TKA [11–14]. Conversely, there are doubts that no improvements exist with the use of PSI [15–18]. Moreover, the entire process to prepare the PSI is a great deal of work, which usually cost several days and thus prolong the hospitalization time of patients [12].

The use of Artificial Intelligence (AI) may solve these problems. In recent years, the application of AI in fields of radiology diagnosis and surgical decision-making shows its potential capabilities [19, 20]. In addition, 3-dimensional (3D) printing technology has become much more mature in fields of every aspect of medicine [21, 22]. Major breakthroughs have been made in PSI design and production thanks to the application of 3D printing based on AI. As a result, an intelligent preoperative planning technique based on preoperative CT images was applied. Based on this planning engine, we designed and produced intelligent-PSI (i-PSI), which were generated and stimulated by AI and produced by 3D printing to best fit patient’s specific anatomy.

Therefore, we conducted this prospective randomized controlled trial to investigate the efficiency of i-PSI in TKA, compared with the conventional instrumentation-TKA (CI). Besides, we also investigate the practicability and security of i-PSI in order to provide references for widespread use of i-PSI. The outcomes of TKA were represented by these proposed indexes: (1) Resection accuracy; (2) radiographic alignment; (3) perioperative period outcomes (clinical outcomes); (4) complications incidence.

## Materials and methods

### Study design

This study was a single-center randomized controlled trial performed in the Department of Joint Orthopedics of The Third Affiliated Hospital of Southern Medical University. This study was approved by the Ethics Committee of the hospital (2021-050), and informed consent was obtained from all individual participants included in this study. To facilitate impartial data collection and analysis of the examined outcomes, both patients and assessors were blinded. This study included patients who underwent TKA at our institution between May, 2022 and July, 2023. The inclusive and exclusive criteria are listed below.

Inclusive criteria: age > 40 and < 80 y; met the diagnostic criteria of osteoarthritis of American Academy of Orthopaedic Surgeons (AAOS) [23]; consent to participate in this study; met TKA indications.

Exclusive criteria: patients who disagreed to participate in this study; inflammatory arthritis; revision TKA; active infections; arthrocentesis in recent 6 mons; poor body condition that might not withstand the surgery.

**Fig. 1:**
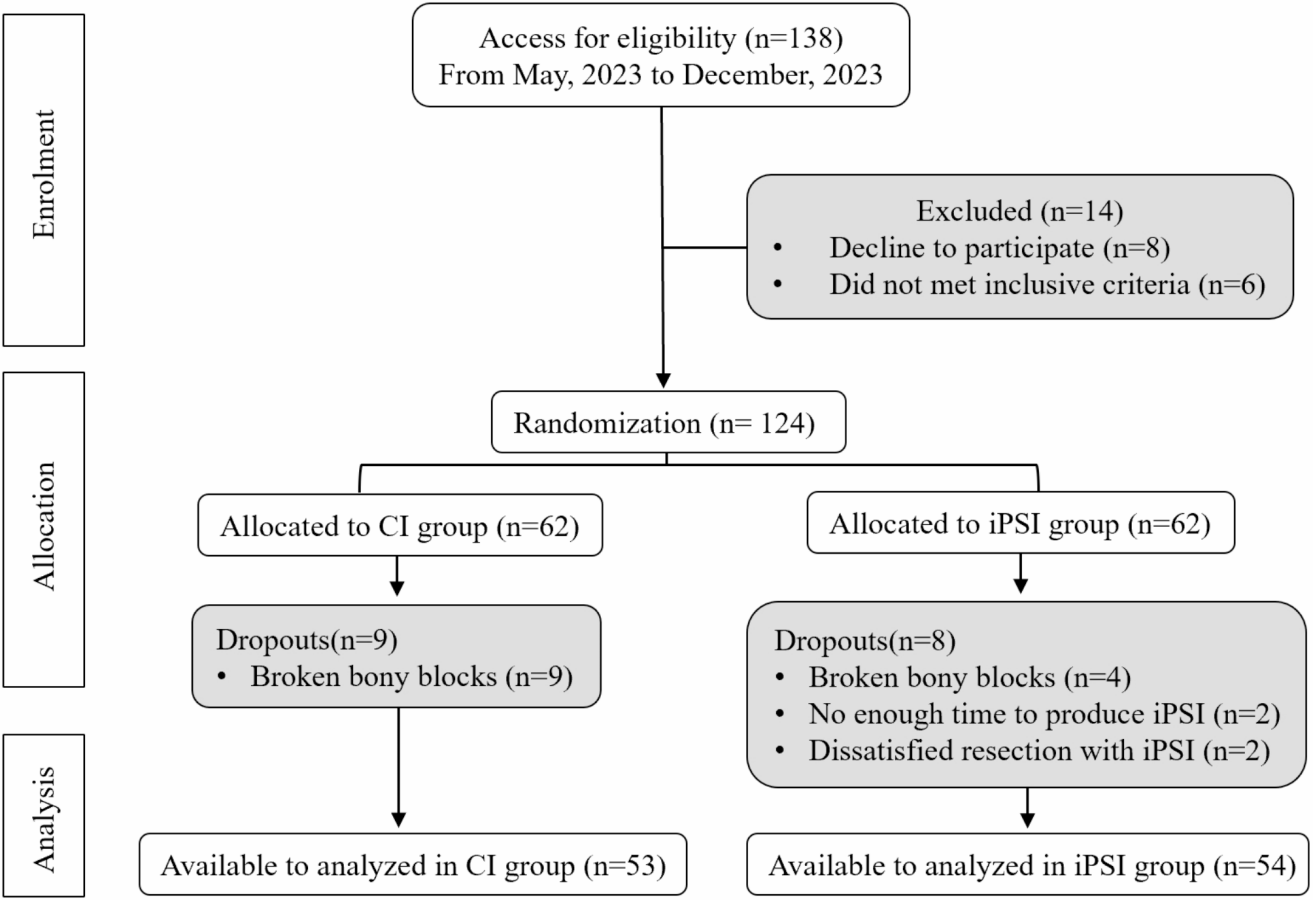
Study flowchart of patient enrollment, allocation and analysis

With computer-generated permuted blocks of four, patients were randomized by stratified blocked randomization, which assessors were unaware of block size. Bilateral procedures were included and randomized once, with both sides being assigned to the same group. Patients were divided into i-PSI group or CI group according to this procedure (Fig. 1).

### Preoperative planning & patient-specific instrument

Both the CI group and the i-PSI group underwent the same CT procedure: full-length lower extremities thin slice CT scans (1 mm). In both groups, CT data was extracted and imported into AI preoperative planning engine AI KNEE (Version 3.0, Longwood Valley Technology, Beijing, China), in which CT image processing, component planning and PSI designing were performed [24]. CT images were first automatically segmented into four parts: femur, tibia, fibula and patella by 3D-UNet, which was developed form convolutional neural networks (CNNs).

Meanwhile, modified High-Resolution Network (HRNet) structures were used to identify featured anatomical landmarks so as to calculate reference lines and angles. After CT segmentation and landmarks identification, a 3D model of the patient’s bone was generated, real-time observation and adjustments of resections, alignments and component positions were allowed. Based on the computed angles and reference lines, the optimal prosthesis was match and the personalized joint simulation results were finalized. So far, surgical decision of the both groups had been made.

Based on each patient’s preoperative planning data and different resection planes, i-PSI could achieve automatic fitting and support real-time adjustments. I-PSI calculates the distance between the bone and the resection guide based on the final resection thickness, ensuring preoperative planning aligns with intraoperative situation. Additionally, when installing on the bone, the screw holes of the PSI and the osteotomy tool are also matched, enabling precise planning and rapid simulation. The i-PSI enabled resection of both the distal femur and proximal tibia and determines the rotation of the femoral component, so the posterior condylar resection could be done without intraoperative remeasurement. Sizes, positions and directions of i-PSI were simulated and customized optimal alignments were documented. After final verification and recognition were gained from two surgeons and one engineer, the data was sent to the Selective Laser Sintering (SLS) Technology 3D printer (Longwood Valley Technology, Beijing, China). Then a primary i-PSI kit was produced by the 3D printer and sent to hydrogen peroxide low-temperature plasma sterilization. With the help of artificial intelligence, the whole procedure was largely simplified and could be completed within a 15-hour timeframe.

**Fig. 2:**
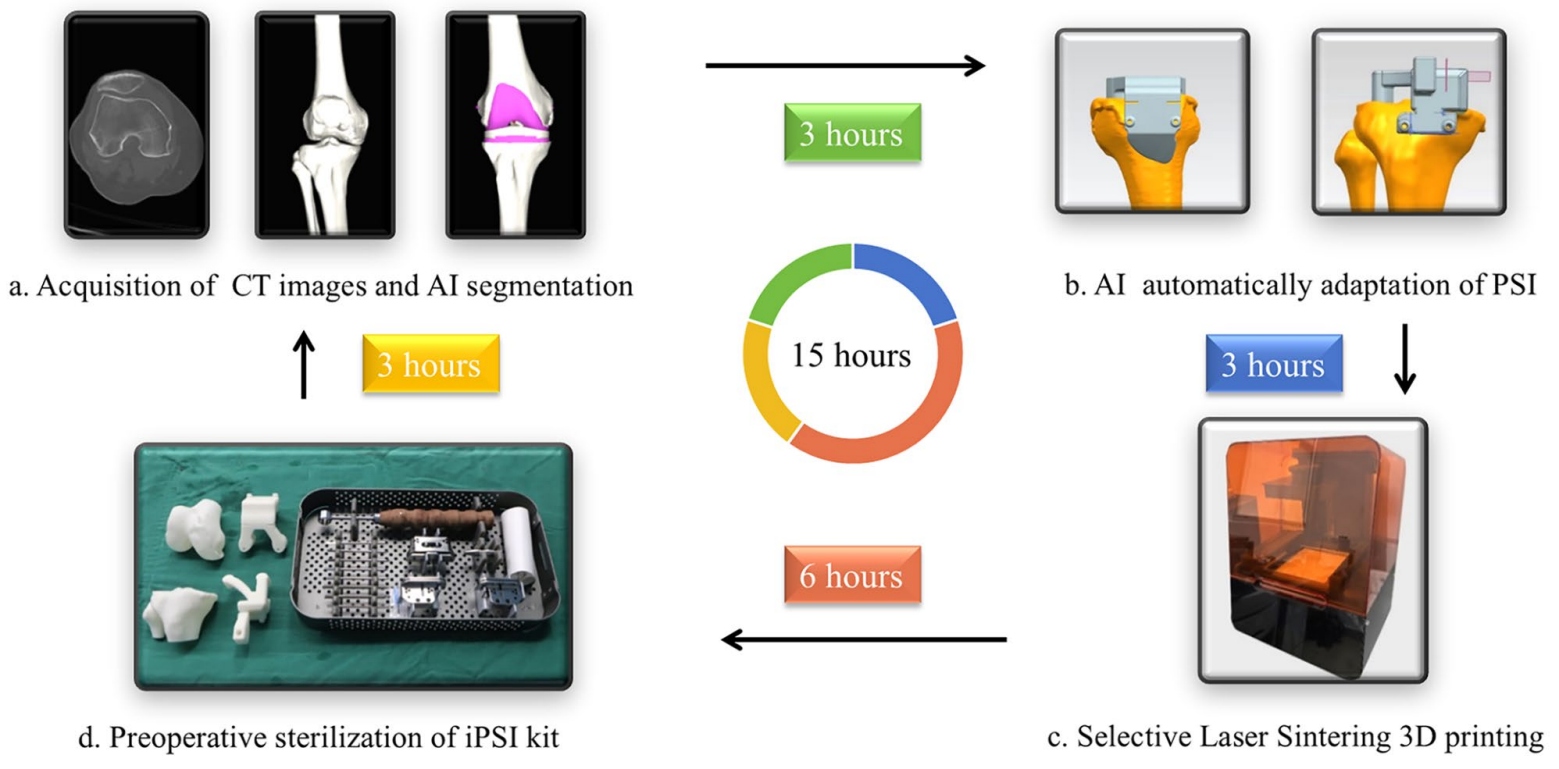
procedures of i-PSI designation and production. (**a**) Acquisition of CT images of patients and automatically segmentation by AI, which totally costs about 3 h. (**b**) Automatically adaptation of i-PSI by AI and confirmed by surgeon before finally being printed, which costs about 3 h. (**c**) Selective Laser Sintering 3D printing for i-PSI entity, which takes about 6 h. (**d**) Preoperative sterilization of i-PSI kit, which costs 3 h

An i-PSI kit was made of polyamide nylon and included four components: models of a distal femur and a proximal tibia, a femoral guide and a tibial guide (Fig. 2). The femur guide was stabilized by three fitting surfaces compacting medial and lateral distal femur and anterior femoral condylar. The tibial guide was stabilized by three fitting surfaces compacting medial and lateral tibial plateau and medial site of tibial tuberosity avoiding hanging and direct contact to the tuberosity. An alignment hole was design to confirm optimal alignment by a conventional extramedullary guide.

### Baseline characteristics

Baseline characteristics included ages, sexes, weights, heights, body-mass-indexes, operative sides, preoperative Hip–knee–ankle axis (HKA axis).

### Surgical techniques & perioperative period management

Every patient underwent TKA through the medial parapatellar approach and received the same kind of Total Knee Systems with posterior-stabilized prostheses and rotating platforms (Attune, DePuy Orthopedics Inc, Warsaw, IN). In all cases, no patella replacement was performed. All surgeries were performed by one proficient surgeon, who possessing more than 5 years of experience in TKA. In both groups, patients were operated with tourniquet and treat with identical pain and blood management protocol. Both groups had same procedures according to routine TKA, except the steps showed below.

In the CI group, following joint exposure, the osteophytes, synovium and fat pads were excised. Then a conventional intramedullary alignment guide and a distal femoral cutting block were used to make a distal femoral cut, setting at 6º of valgus. Whilst a proximal tibia cut was made by using an extramedullary alignment guide and a tibial cutting block. After verifying femoral size and rotation, the A/P Chamfer Block that matched the femur size was placed and resection of the anterior and posterior femoral condylar were performed.

**Fig. 3:**
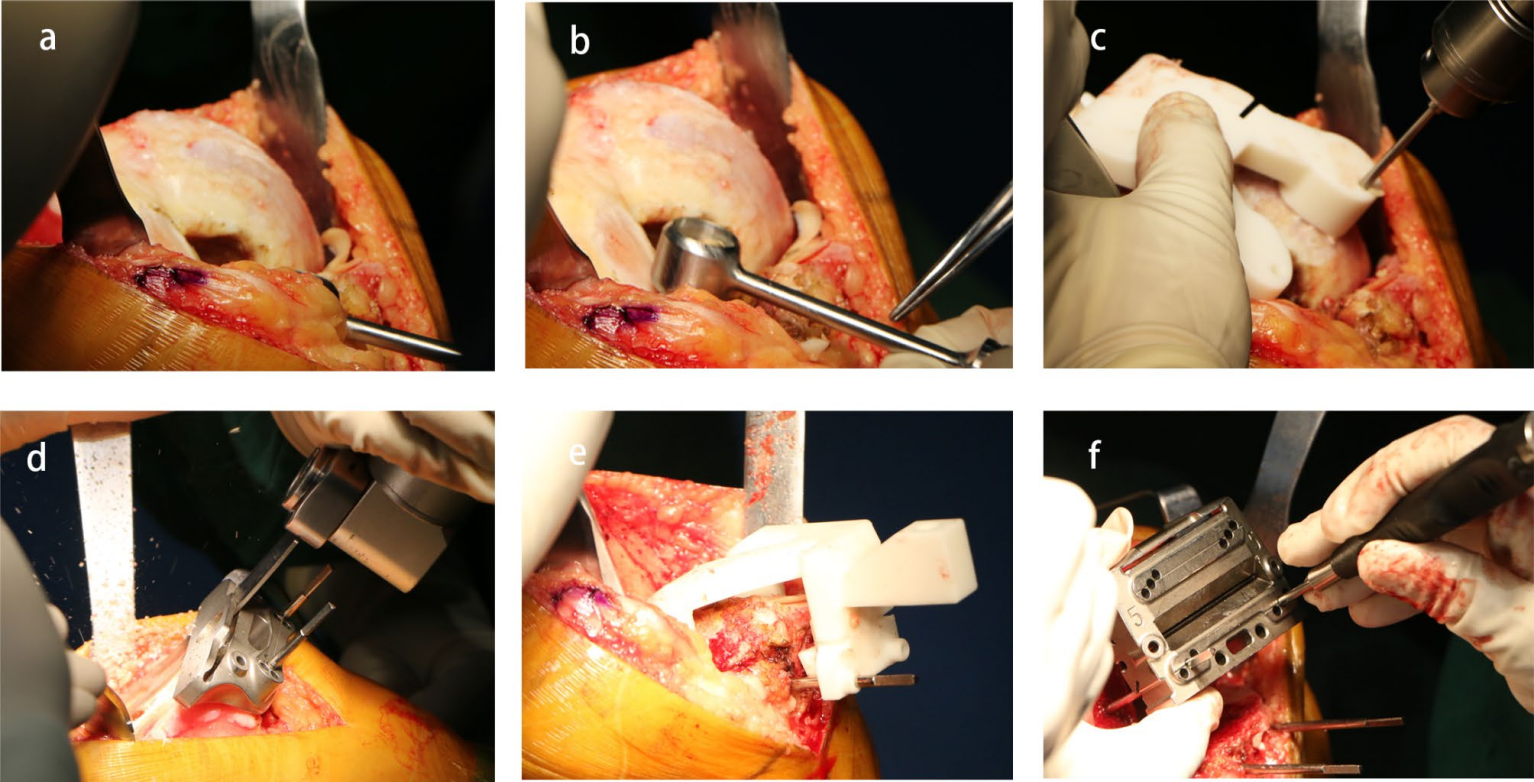
Surgical procedure of i-PSI group. (**a**) Incision and exposure of joint cavity. (**b**) Femoral cartilage removal. (**c**) Installation of femoral component by four pins, two for distal demur and two for anterior cortex. (**d**) Distal femur resection. (**e**) Same procedure of Tibia. (**f**) A/P Chamfer Block were fixed by two pins in the two pin holes previously drilled in Step(c)

In the i-PSI group, following joint exposure and tissues excision, the cartilage of the distal femoral condylar was removed and then the i-PSI was installed on the subchondral bone and pinned, resection of the distal femoral condylar was performed under i-PSI guidance. The same resection technique was performed on tibial plateau. The A/P Chamfer Block, matching to the remaining pin holes in the distal femur, was inserted using two pins and followed by the resection of the anterior and posterior femoral condyles. It is noted that the i-PSI was used only for orientation, resection was still performed by conventional cutting blocks (Fig. 3).

**Fig. 4:**
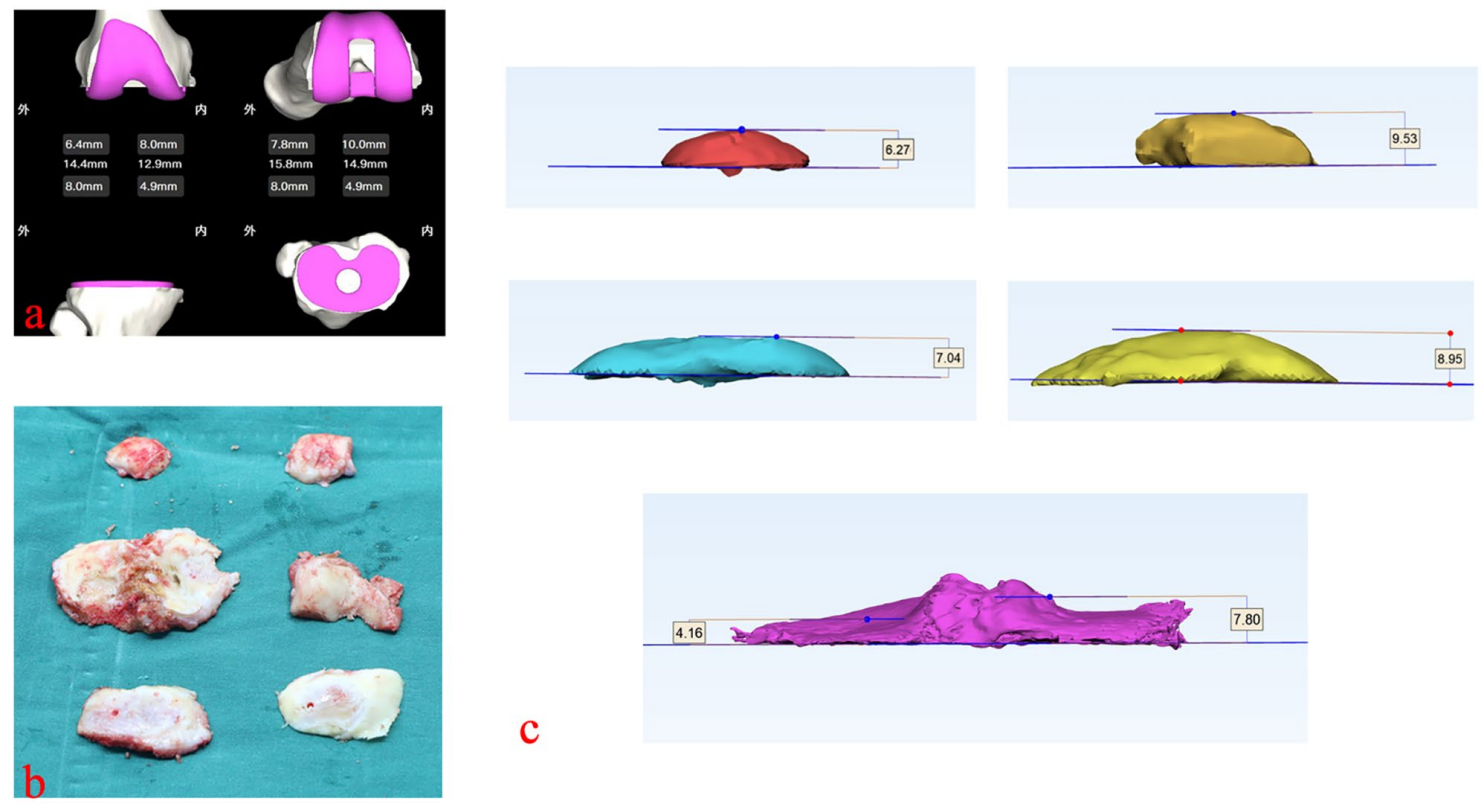
Resection block measurement. (**a**) Preoperative planning of resection thicknesses. (**b**) Intraoperative collection of bony blocks. (**c**) Postoperative CT measurements of thicknesses of bony blocks

During the operating procedure, both CI and i-PSI group’s actual data of bony resection were recorded and compared with the preoperative one. Deviation is defined as the actual thickness minus preoperative planning thickness. Negative numbers indicate inadequate resection (undercut), while positive numbers mean excessive resection (overcut). To account for the additional bone lost due to the thickness of the saw blade, 1.19 mm was added to the measurement (Fig. 4). Resection ratio difference: referring to the difference between the lateral resection deviation and the medial deviation on the same resection plane, including the distal femur and tibial plateau planes (the posterior femoral condyles are not compared here due to the difficulty of medial-lateral comparison on the flexion knee). The resection ratio difference is equal to the lateral resection deviation minus the medial resection deviation. In the same resection plane, a positive result indicates a relatively greater residual resection amount on the medial side, while a negative result indicates a greater residual resection amount on the lateral side. This metric may impact postoperative alignment: a greater residual resection amount on the lateral side may result in postoperative varus alignment, while a greater residual resection amount on the medial side may result in postoperative valgus alignment. Relationships between postoperative alignment and resection ratio differences were analyzed by correlation analysis.

### Radiographic measurements

**Fig. 5:**
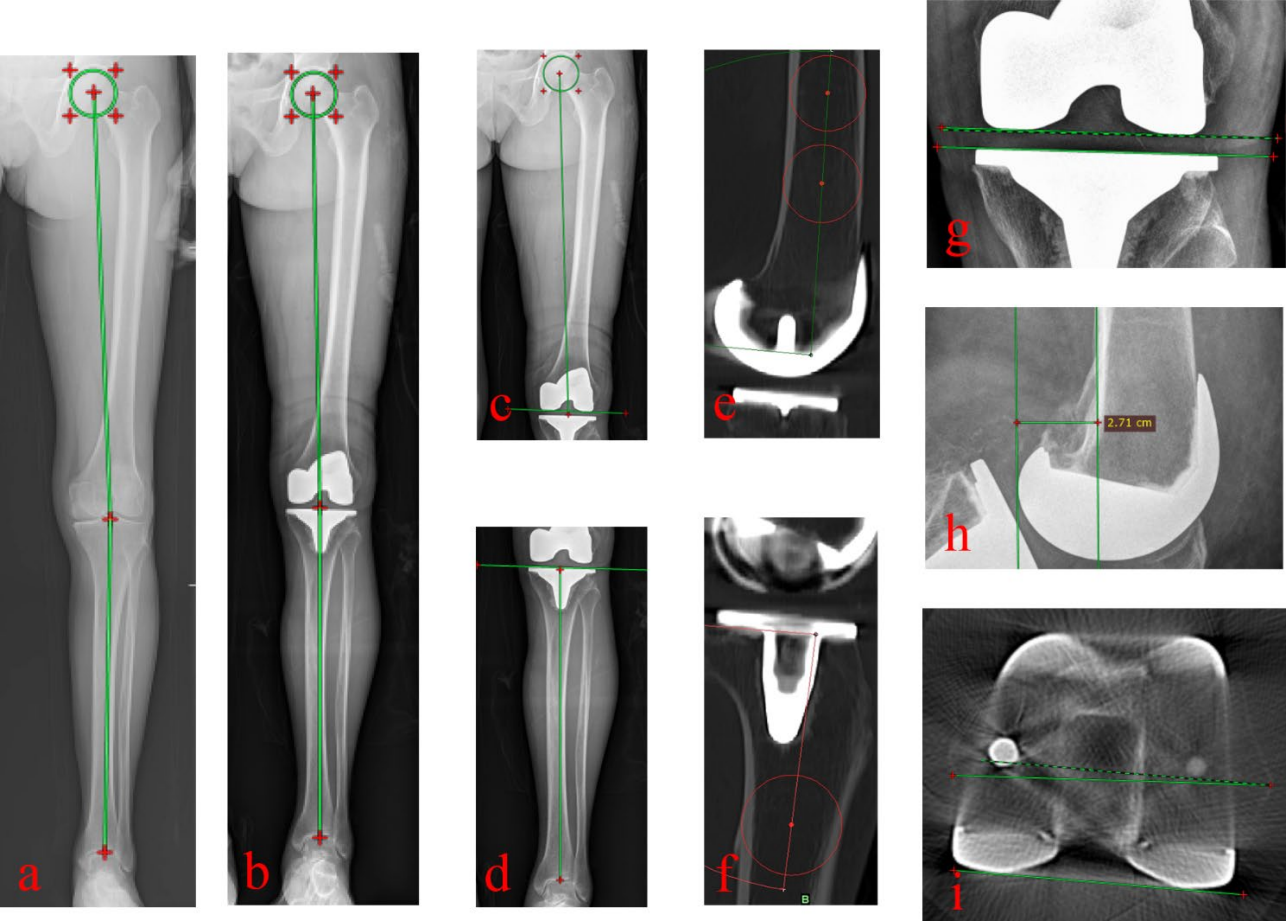
Radiology measurement (**a**) Representative image of pre-operative TKA (**b**) Representative image of post-operative TKA (**c**) Representative image of FCA (**d**) TCA (**e**) Representative image of FSA (**f**) Representative image of TSA (**g**) Representative image of JLCA (**h**) Representative image of PCO (**i**) Representative image of PCA

Each patient performed x-rays images of weight-bearing double lower extremities full-length radiography and short AP & LAT films of the affected knees, both preoperatively and postoperatively. Each patient performed postoperative CT of the surgical side. Measurements of the radiographic outcomes are mostly based on these x-rays and CT images (Fig. 5). The measured alignments and their safe zones were listed below:

Hip–knee–ankle axis (HKA axis): The mechanical axis of the lower limb is calculated as the angle between the femoral and tibial mechanical axes, which is generally aimed at 180˚. The safe zone of HKA is considered between varus or valgus of 180˚±3° [25].

Femoral coronal alignment (FCA): The angle which is constructed by the tangent to the distal femoral condyles and the anatomical axis of the femur and usually considered 90˚±3° for neutral placement [26].

Tibial coronal alignment (TCA): The angle which is constructed by the tangent to the proximal tibial base plate and the anatomical axis of the tibia and usually considered 90 ˚ for neutral placement, 90˚±3° were set as safe zone.

Joint line convergence angle (JLCA): was formed by the tangential lines between the most distal points of the medial and lateral femoral and between the deepest points of the medial and lateral tibial plateau, 0˚±2° were set as safe zone.

The posterior condylar offset (PCO) is the maximal thickness of the posterior femoral condyle projected posteriorly to the tangential of the posterior cortex of the femoral shaft; it has to be maintained after TKA [27]. ΔPCO = preoperative PCO - postoperative PCO.

Femoral sagittal alignment (FSA): the angle which is constructed by the mechanical axis of the femur in the sagittal plane and the tangent of the distal portion of the femoral component. Femoral sagittal alignment = 90 – FSA (+ component flexion, - component extension). Safe zone of FSA is -3°–3° [28].

Tibial sagittal alignment (TSA): the angle which is constructed by the anatomical axis of the tibial in the sagittal plane and the line joining the anterior and posterior point of the tibial component. Tibial slope = 90 – TSA (+ posterior slope, - anterior slope). Safe zone of tibial slope is 0–7° [26, 29].

The posterior condylar angle (PCA): the angle formed by the posterior condylar line (PCL), which tangent to the posterior-most aspect of the femoral component, and the surgical transepicondylar axis (sTEA), which connecting the sulcus of the medial epicondyle and the tip of the lateral epicondyle. Safe zone of PCA were considered external rotation 2–5 ˚ [26, 29].

### Clinical outcomes

Clinical outcomes included surgical time, estimated blood losses and hemoglobin losses. The Complications were also documented. The patient’s estimated blood volume was calculated by the formula brought up by Nadler et al [30]; The patient’s estimated blood loss was calculated by the formula brought up by Gross, J.B [31]. No blood transfusion was applied in any patient.

### Statistical analysis

Continuous variables were summarized as either means and standard deviations or medians with interquartile ranges. Continuous variables were analyzed by Two-sample t test when the variable was distributed normally, and by Mann–Whitney U test when the variable was not normally distributed. Categorical variables were assessed using the Chi-squared or Fisher’s exact test. Relationships between postoperative alignment and resection ratio differences were analyzed by Pearson analysis when the variable was distributed normally, and by Spearman analysis when the variable was not normally distributed. The correlation coefficient (r) assumes any value from − 1 to 1, with an |r| value of less than 0.4 being considered a weak correlation, moderate correlations when |r| is between 0.4and 0.7, and strong correlations when |r| is more than 0.7. Statistical analysis was performed using SPSS version 26 (IBM, Armonk, NY, USA) and GraphPad Prism version 8 (GraphPad Software, La Jolla, CA). Statistical significance was set at values of *P* < 0.05. An a priori sample size calculation was performed in Power Analysis and Sample Size Software 2021 (PASS 2021) based on an anticipated effect size of (δ = 1, σ = 2), a desired statistical power of (Power = 0.8), and a significance level of (Alpha = 0.05).

## Results

### Baseline characteristics

In total, there were 107 eligible knees assigned to the group of TKAs using patient-specific instruments (*n* = 54) or to the group of TKAs using conventional instruments (*n* = 53). All the baseline characteristics showed no significant differences between the CI group and the i-PSI group (*p* > *0.0.5*). See Table 1 for details.

**Table 1:** Baseline characteristics of two groups

characteristic	CI	i-PSI	*P* value
Patients, *n*	53	54	
Ages, y	67.8 ± 7.7	68.6 ± 7.1	0.629
Sexes, male%	13 (24.5%)	9 (16.7%)	0.314
Sides, left%	25 (47.2%)	24 (44.4%)	0.777
BMI, kg/m²	26.3 ± 3.7	26.1 ± 3.4	0.782
HKA, °	9.1(5.7, 12.25)	8.2 (3.85,12.35)	0.543
JLCA, °	5.2(2.8, 7.5)	6.1 (2.8,8.4)	0.370

HKA, JLCA direction: positive numbers represent varus, negative numbers represent valgus

### Resection outcomes

The Deviations of lateral distal femoral condyle of CI group was − 0.72 (-1.31, -0.13) mm and − 0.38(-0.79, 0.10) mm of i-PSI group, which met statistical significance (*P* = 0.032). The Deviations of medial distal femoral condyle of CI group was (-0.58 ± 0.98) mm and (-0.21 ± 0.78) mm of i-PSI group, which met statistical significance (*P* = 0.035). No significant differences were found in other resection outcomes of the two groups. The detailed results are shown in Table 2.

**Table 2:** Differences of resection outcomes of the two groups

	CI	i-PSI	*P* value
Lateral distal femoral condyle, mm	-0.72 (-1.31, -0.13)	-0.38(-0.79, 0.10)	0.032
Medial distal femoral condyle, mm	-0.58 ± 0.98	-0.21 ± 0.78	0.035
Distal condyle resection ratio difference, mm	0.02 ± 1.07	-0.14 ± 0.78	0.513
Lateral posterior femoral condyle, mm	-0.79 ± 1.30	-0.42 ± 1.33	0.152
Medial posterior femoral condyle, mm	-0.42 ± 1.17	-0.56 ± 1.29	0.572
Posterior condyle resection ratio difference, mm	-0.37 ± 1.58	0.13 ± 1.32	0.078
Proximal Lateral plateau, mm	-0.44 ± 1.17	-0.24 ± 0.95	0.355
Proximal Medial plateau, mm	-0.69 ± 1.18	-0.40 ± 0.94	0.169
Proximal plateau resection ratio difference, mm	0.27 ± 1.35	-0.17 ± 0.94	0.053

Deviations: negative numbers indicate inadequate resection (undercut), while positive numbers indicate excessive resection (overcut). Ratio differences: negative number indicate varus/internal rotation, while positive numbers indicate valgus/external rotation

### Radiography outcomes

In coronal plane, the postoperative HKA in CI group was 1.60 (-0.95, 2.80) ° varus and 0.60 (-1.15, 1.33) ° varus in i-PSI group, which met statistical significance (*P* = 0.025). The postoperative HKA outliners in CI group was 14 (26.4%) and 6 (11.1%) in i-PSI group, which met statistical significance (*P* = 0.042). The FCA in CI group was (0.61 ± 1.87) ° valgus and (0.24 ± 1.84) ° varus in i-PSI group and met statistical significance (*P* = 0.019). The JLCA in CI group was (0.56 ± 1.16) °varus and (0.14 ± 0.95) °varus in i-PSI group and also met statistical significance (*P* = 0.043). In sagittal plane, the FSA in CI group was (0.91 ± 2.80) ° extension and (0.56 ± 2.52) ° flexion in i-PSI group, which met statistical significance (*P* = 0.005). No significant differences were found in other radiological outcomes. The detailed results are shown in Table 3. There was no statistically significant correlations between postoperative alignment and resection ratio differences. The detailed results are shown in Table 4.

**Table 3:** Radiography outcomes of two groups

	CI	i-PSI	*P* value
Coronal alignments			
HKA, °	1.60 (-0.95, 2.80)	0.60 (-1.15, 1.33)	0.025
HKA outliner, *n*	14 (26.4%)	6 (11.1%)	0.042
FCA, °	-0.61 ± 1.87	0.24 ± 1.84	0.019
FCA outliner, *n*	7 (13.2%)	6 (11.1%)	0.740
TCA, °	0.74 ± 2.18	0.15 ± 2.14	0.178
TCA outliner, *n*	9 (17.0%)	11 (20.4%)	0.653
JLCA, °	0.56 ± 1.16	0.14 ± 0.95	0.043
JLCA outliner, *n*	3 (5.6%)	8 (15.1%)	0.104
Sagittal alignments			
FSA, °	-0.91 ± 2.80	0.56 ± 2.52	0.005
FSA outliner, *n*	16 (30.2%)	14 (25.9%)	0.624
TSA, °	2.09 ± 1.62	2.36 ± 2.18	0.476
TSA outliner, *n*	5 (9.4%)	7 (13.0%)	0.563
ΔPCO, mm	-0.08 ± 0.25	-0.03 ± 0.24	0.276
Notching, *n*	Grade I/II 5/3	Grade I/II 6/4	0.632
Transvers alignments			
PCA, °	-2.90(-3.50, -1.96)	-3.00(-3.63, -2.08)	0.581
PCA outliner, *n*	13 (24.5%)	10 (18.5%)	0.449

HKA, FCA, TCA and JLCA direction (Coronal plane): positive numbers represent varus, negative numbers represent valgus. FSA, TSA direction (Sagittal plane): positive numbers represent flexion, negative numbers represent extension. PCA direction (Transvers plane): positive numbers represent interior rotation, negative numbers exterior rotation

**Table 4:** Correlation between postoperative alignment and resection ratio differences

		FCA	TCA	HKA	JLCA
Distal femoral resection ratio difference	*p*	0.111	\	0.353	0.106
	*r*	-0.155	\	-0.091	0.157
Proximal tibial resection ratio difference	*p*	\	0.646	0.691	0.508
	*r*	\	-0.045	0.039	-0.065
Coronal resection ratio difference	*p*	0.810	0.202	-0.795	0.638
	*r*	-0.024	-0.124	-0.025	0.046

### Clinical outcomes

The surgical time was 76.0(71.0, 83.5) minutes in the CI group and 81.9(73.8, 91.8) minutes in the i-PSI group, which met statistical significance (*P* = 0.027) (Table 5). The estimated blood losses, hemoglobin losses and incidences of complications between the two groups show no statistical significance. During hospitalization, one patient experienced poor wound healing, after undergoing debridement of the soft tissue, the wound healed well with no further exacerbation of infection.

**Table 5:** Clinical outcomes

	CI	i-PSI	*P* value
Surgical time, min	76.0(71.0, 83.5)	81.9(73.8, 91.8)	0.027
Estimated blood volume, ml	3,930.0 ± 567.4	3,845.9 ± 509.6	0.421
Estimated blood losses, ml	645.7(397.8,782.6)	561.9(317.9,769.0)	0.501
Hemoglobin losses, g/L	18.0(11.5,24.5)	16.5(9.0,23.0)	0.495
Postoperative complications,	1 (1.9%)	0	0.993

## Discussion

Ever since the day when patient specific instrumentation came out, debates of whether application of PSI result in better outcomes than conventional instrumentation has come along [28, 32–34]. Despite variations in protocols and production processes among different PSI systems, studies of clinical practice of PSI have been started in different health centers all over the world. Some of which concentrate on radiological outcomes [18, 34], while others focus on clinical or surgical outcomes [34–38]. In this study, we emphasized in simplifying the procedure of PSI with artificial intelligence, while at the same time investigating the bony resection accuracy and radiological outcomes of the i-PSI.

For resection accuracy, we concluded that there was enhanced precision of distal femur resection in i-PSI group compared to the CI group, showing the particular strength of i-PSI in this aspect. Enhanced accuracy of resection may result in better outcomes, including a reduced risk of aseptic loosening and other associated complications [26, 39–42]. However, resection accuracy of posterior femoral condyle or tibial plateau showed large tendency of dispersion in both groups, representing less accuracy compared with the distal femur resection, which in lines with previous research [35–37]. In addition, no statistical differences were found in the resection accuracy of posterior femoral condyle or tibial plateau between the two groups. This may because when proceeding the distal femoral resection, the anterior Universal Pins remained fixed and the distal Universal Pins needed to be removed, so resection of the distal femur was directly guided by fixed anterior Universal Pins. When it comes to the A/P and Chamfer Cuts, the removed distal Universal Pins needed to be repined manually, which may deviate from the planned position and cause discrepancy. No statistical differences were found in the resection ratio difference, too. The discrepancy between these results may be related to the inadequate sample size, different intraoperative balance technique and precision of the i-PSI kit. Overall, the resection tendency of i-PSI group was to undercut, as a result of the conservative setting of preoperative planning system to ensure controllability and safety, which is in accordance with the overall tendency of the reported PSI system.

For radiographic alignments, we found that i-PSI has the potential to enhance the alignment of the TKA components. The results showed enhanced accuracy in coronal alignment such as postoperative HKA, HKA outliners, FCA and JLCA, compared with the CI group. Each of these coronal parameters (except TCA) in i-PSI group showed closer position to neutral alignment, especially HKA, FCA and JLCA. Neutral postoperative alignment has been seen as one of the major objects in mechanical alignment [41–43]. However, the sagittal alignments between the two groups showed no differences. Perhaps, the sagittal alignments were affected by various factors, such as unstable radiography positions that easily deviated from standardized radiography position, impacting the measurement of sagittal parameters. Otherwise, the gap between i-PSI and CI didn’t seem to be large enough to be detected by present methods in this study.

Surprisingly, no correlation was found between the resection ratio difference and the postoperative alignment. The results imply that while bony resection is a necessary condition, it may not be sufficient to solely determine postoperative alignments. After all, different soft tissues conditions, gap balance skills or measurement errors could influence this result. Therefore, the reliability of resection ratio difference as a predictor for postoperative alignment requires further verification.

For perioperative period outcomes and complications incidences, the surgical time of i-PSI group was longer than CI group and met statistical differences, which was similar in other studies [15, 32]. The primary factor prolonging time might be caused by the step of scraping cartilage. Scraping cartilage evenly confronts challenges in patients with severe damaged cartilage surfaces or conversely, those with relatively thick cartilage surface. Additionally, when i-PSI did not compact firmly on the bone, adjustments including soft tissue removal and body position changes were necessary and cost extra time to solve this problem. However, no differences were found in other clinical outcomes between the two groups.

With the investigation of i-PSI in this study, we conclude several superiorities of i-PSI compared with CI. i-PSI showed better accuracy in distal femur resection and better coronal alignments than CI, which suggested i-PSI was a practical tool in TKA. Additionally, for patients with deformative femurs or previous femoral internal fixation, intramedullary guide might cause fracture or mal-alignment, i-PSI not only addresses this issue but also achieved more accurate resection. We also speculate that of inserting an intramedullary guide could result in reduced trauma, thus result in less blood or hemoglobin losses. However, due to short-term follow-up, no differences were found in the clinical outcomes between the two groups.

Meanwhile, i-PSI showed some advantages compared with traditional manual PSI. First of all, previous studies showed that PSI production was a complex and time-consuming job, which is both costly and cumbersome [12]. As an AI-driven and 3D-printed tool, simplified production steps have shortened the production time to 15 h. At the same time, accuracy and security were reserved. Secondly, in AI KNEE, the automatic fitting of PSI, compared to traditional manual fitting, offers the advantage of automatically aligning to the optimal position, while allowing for real-time personal adjustments so as to adapt flexibly. Moreover, AI algorithms ensure consistency in identification results, unaffected by human factors such as mood or fatigue, whereas manual identification may be subjective. AI models can continuously improve through ongoing learning. As the amount of training data increases and algorithms are optimized, the performance will progressively enhance. Therefore, we believed that i-PSI has the potential to shorten the hospital stay and providing convenience for patients.

Nevertheless, some drawbacks of i-PSI were found in this study. First, patients in i-PSI group were exposed to extra radiation doses due to the acquisition of extra CT scans, compared with routine knee joint radiography in the CI group. Secondly, less experienced surgeons might be unable to adjust deftly the preoperative settings of the resection plan, as a result they might be misguided and resect mistakenly. Thirdly, the result showed that i-PSI TKA required more time than CI TKA, which could increase the risk of complications from tourniquet. Forth, for patients with severe knee deformities, merely relying on bony resection alone is insufficient to correct knee joint deformities. Intraoperatively appropriate soft tissue release may be required, necessitating surgeons to acquire proficiency based on their own experience. Finally, patients might rather choose conventional TKA than i-PSI TKA because of increased cost.

Besides, the AI procedures of i-PSI face following limitations: Like other artificial intelligence systems, our software requires extensive training data and optimization. In the medical field, obtaining a large quantity of high-quality and accurately marked medical imaging data is challenging, which restricts and delays the training effectiveness and evolutionary capability of our software. Furthermore, when handling complex and highly variable cases, such as knee joint ankylosis, the software may struggle to recognize specific points and data, thus impacting the accuracy of resection planning.

This study incorporates a degree of innovation in its design. Previous research generally used vernier calipers for manual measurements of the resection thickness [36]. However, it is reported that measurement by vernier calipers face several problems, including interference of the cartilage layer of the bony block and the concave anatomical shape of tibia plateau which is difficult to measure. CT measurement of the bony block might solve this problem by avoiding the influence of the cartilage layer [35]. Moreover, we maintain consistency in the measurement procedure for the cutting bones as in preoperative CT planning, which may maximize the comparability between these two types of data. Although there existed no correlation between the resection ratio difference and the postoperative alignment, it is still the first time this novel parameter being introduced. This parameter was set to represented intraoperative bone resection thickness of two different compartment in the same plane, consequently affecting postoperative alignment. Given the scarcity of related research and the negative result of this study, further verification is necessary, and it still holds a certain degree of research value.

There are also certain limitations in this study. First of all, it principally focuses on intraoperative validation of i-PSI and immediate postoperative radiographic outcomes, while leaves a notable gap of deficiency of functional analysis and long-term follow-up. The absence of these elements hindered the comprehensive assessment of i-PSI. Thus, the different functional outcomes between the two groups, resulting from differences in resection accuracy and radiological outcomes, remained further verification. Secondly, there were a few drop-outs in the two groups. Intraoperative broken bony blocks were unmeasurable, unavoidable and unsolvable, which may lead to bias because of the drop-out information. Thirdly, only one kind of prostheses was used in this study and no patella arthroplasty was operated. This restriction might limit the general applicability of i-PSI to different protheses or various operative procedure. Fourth, the baseline character of our study has a preponderance of overweight women, potentially reducing the representativeness of this sample for other baseline characteristics and resulting in selection bias. Fifth, no postoperative ultrasound examinations were conducted, leading to misdiagnosis of subclinical VTE in postoperative patients, resulting in data bias. Finally, all measurements were completed by one single observer, so that no interobserver comparisons could be calculated and might lead to measurement bias.

## Conclusion

The i-PSI did provide improved resection accuracy, more neutral synthesis position and limb alignment than CI. The superiorities of i-PSI, including practicality, precision, and efficiency, states that i-PSI being an alternative tool in TKA, indicating promising prospects for its clinical application.

## Data Availability

The data that support the findings of this study are available from the corresponding author, Chang Zhao, upon reasonable request.

## Declarations

### Permission to reproduce material from other sources

No material from other sources were reproduced.

## Abbreviations

TKA: Total knee arthroplasty
AI: Artificial intelligent
CI: Conventional instrumentation
i-PSI: Intelligent patient-specific instrumentation
3D: 3-Dimension
CNNs: Convolutional neural networks
SLS: Selective laser sintering
HRNet: High-resolution network
BMI: Body-mass-indexes
HKA: Hip–knee–ankle axis
FCA: Femoral coronal alignment
TCA: Tibial coronal alignment
JLCA: Joint line convergence angle
PCO: Posterior condylar offset
FSA: Femoral sagittal alignment
TSA: Tibial sagittal alignment
PCA: Posterior condylar angle
PCL: Posterior condylar line
sTEA: Surgical transepicondylar axis
